# Modular pathway engineering for enhanced production of *para*-aminobenzoic acid and 4-amino-phenylalanine in *Escherichia coli* via glucose/xylose co-utilization

**DOI:** 10.1128/aem.02468-24

**Published:** 2025-04-17

**Authors:** Daisuke Nonaka, Mayumi Kishida, Yuuki Hirata, Ayana Mori, Akihiko Kondo, Yutaro Mori, Shuhei Noda, Tsutomu Tanaka

**Affiliations:** 1 Department of Chemical Science and Engineering, Graduate School of Engineering, Kobe University 542048, Kobe, Hyogo, Japan; 2 Graduate School of Science, Technology and Innovation, Kobe University 592891, Kobe, Hyogo, Japan; 3 PRESTO, Japan Science and Technology Agency (JST) 13501, Kawaguchi, Saitama, Japan; Danmarks Tekniske Universitet The Novo Nordisk Foundation Center for Biosustainability, Kgs. Lyngby, Denmark

**Keywords:** parallel metabolic pathway engineering, *p*-aminobenzoic acid, 4-amino-phenylalanine, modular metabolic engineering, *Escherichia coli*

## Abstract

The modularization of biosynthetic pathways is a promising approach for enhancing microbial chemical production. We have developed a co-utilization method with glucose and xylose substrates to divide metabolic pathways into distinct production and energy modules to enhance the biosynthesis of *para*-aminobenzoic acid (pABA) in *Escherichia coli*. Optimizing initial glucose/xylose concentrations and eliminating carbon leakage resulted in a pABA titer of 8.22 g/L (yield: 0.23 g/g glucose). This strategy was then applied to the biosynthesis of 4APhe, a compound synthesized from chorismate without pyruvate (PYR) release. Utilizing glucose and xylose as co-substrates resulted in the production of 4.90 g/L 4APhe. Although 4APhe production did not benefit from PYR-driven energy generation as pABA production did, high titer was still achieved. This study highlights the effectiveness of modular metabolic pathway division for enhancing the production of key aromatic compounds and provides valuable insight into microbial production of chemicals that require specific biosynthetic donors such as amino groups.

**IMPORTANCE:**

Microbial biosynthesis of chemicals from renewable resources offers a sustainable alternative to fossil fuel-based production. However, inefficiencies due to substrate diversion into by-products and biomass hinder optimal yields. In this study, we employed a modular metabolic engineering approach, decoupling pathways for chemical production from cell growth. Using glucose and xylose as co-substrates, we achieved the enhancement of *p*-aminobenzoic acid production in *Escherichia coli*. Additionally, we demonstrated the versatility of this approach by applying it to the biosynthesis of 4-amino-phenylalanine production. This study highlights the potential of modular metabolic pathway division for increased production of target compounds and provides valuable insight into microbial production of chemicals that require specific biosynthetic donors such as amino groups.

## INTRODUCTION

*p*-Aminobenzoic acid (pABA) is utilized as a crosslinking agent for resins, as a component in dyes and feedstocks, and as a precursor molecule in the pharmaceutical industry (1, 2). Furthermore, biosynthesis of pABA is a promising alternative supply route for the production of polyethylene terephthalate (PET) and *p*-phenylenediamine (3). The global demands of both PET and *p*-phenylenediamine are expected to rise in the future; thus, biotechnological production of pABA could represent a vital, sustainable alternative.

Advances in metabolic engineering and synthetic biology have led to the development of a range of tools and strategies for constructing efficient microbial cell factories (4–8). An engineered strain of *Escherichia coli* with the expression of *pabAB* from *Corynebacterium efficiens* exhibited higher pABA production compared to those with the endogenous *pabAB* (9). An engineered strain of *C. glutamicum* overexpressing exogenous feedback-resistant aroG^S180F^ gene, *pabAB*, and *pabC* produces 6.2 g/L of pABA (10). Increasing chorismate availability by eliminating the synthetic pathways of aromatic amino acids and partially removing phosphoenolpyruvate (PEP)-consuming pathways has been reported to further increase pABA production in engineered *E. coli*, up to 2.46 g/L of pABA (11).

The metabolic burden imposed by genetic or environmental alterations on host cells presents a significant challenge to microbial engineering (12), often leading to impaired growth and suboptimal metabolic function (13). The modularization of synthetic pathways has revealed innovative strategies for the systematic optimization of engineered microbial strains. In modular metabolic engineering, enzymes within pathways are organized into distinct, interacting modules to restore metabolic balance and enhance efficiency (14–16). We have previously restructured the native metabolic pathway of *E. coli* to funnel all produced PEP into the biosynthesis of aromatic compounds (17). The production module consisted of glycolysis and the pentose phosphate pathway and was separated from the energy module at the point of the PEP-to-pyruvate (PYR) conversion. To address the lack of PYR, we further developed a parallel metabolic pathway engineering (PMPE) strategy to restore PYR supply and optimize cell growth (18). To this end, we engineered a xylose catabolic pathway, which directly integrates with the tricarboxylic acid (TCA) cycle. This circumvents interference with glycolytic and pentose phosphate pathways, allowing glucose to be primarily allocated to the synthesis of desired chemicals, while xylose is utilized to support cell proliferation. The strain that was engineered for PMPE demonstrated significant improvements in production (18, 19).

**Fig 1:**
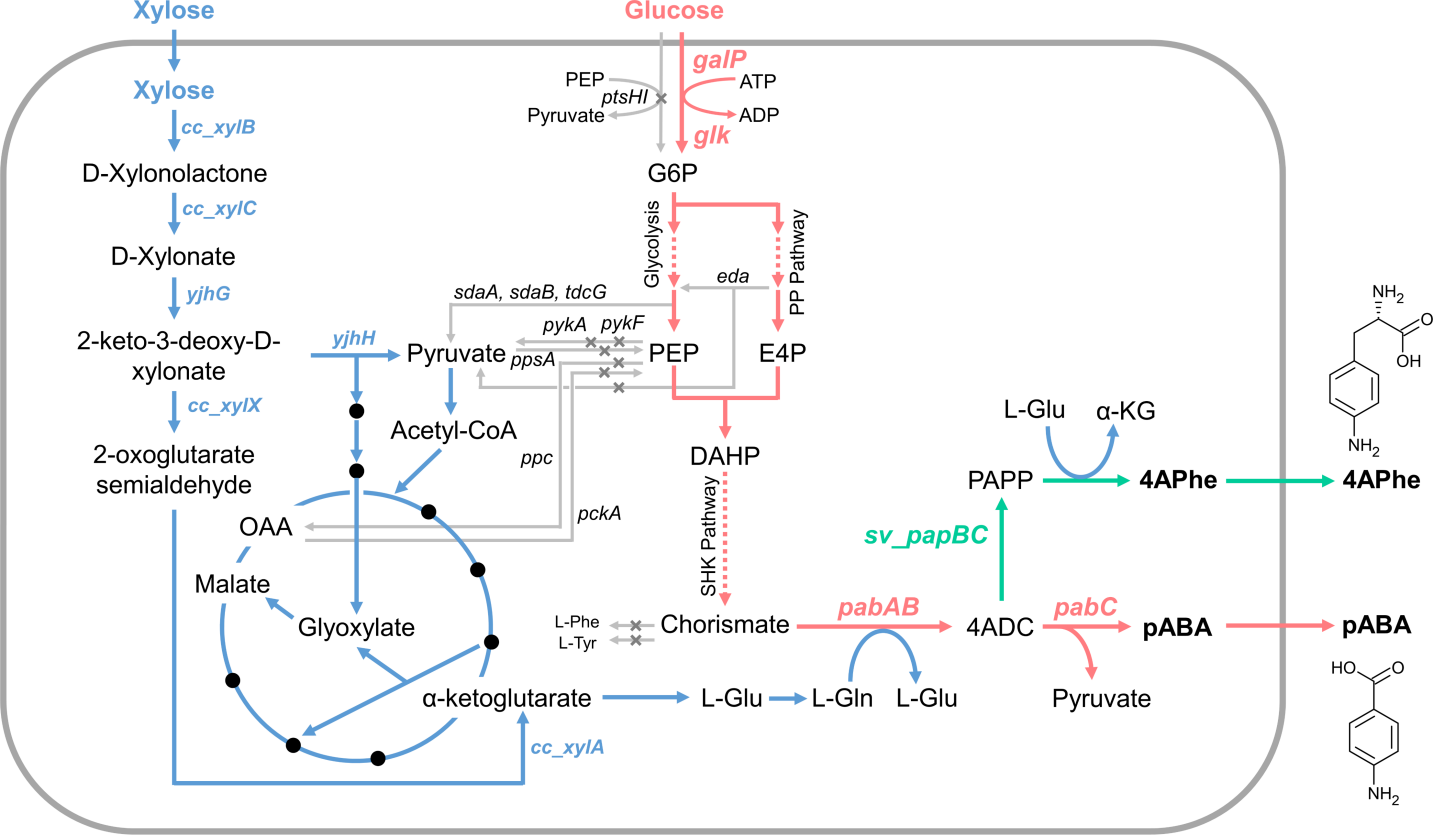
Metabolic engineering of the *Escherichia coli* strain for production of *p*-aminobenzoic acid (PABA) or 4-amino-phenylalanine (4APhe). Gray crosses indicate deletions of genes in the GX1 strain. 4ADC, 4-amino-4-deoxychorismate; DAHP, 3-deoxy-D-arabino-heptulosonic acid 7-phosphate; E4P, erythrose 4-phosphate; G6P, glucose 6-phosphate; L-Gln, L-glutamine; L-Glu, L-glutamate; L-Phe, L-phenylalanine; L-Tyr, L-tyrosine; PAPP, 4-aminophenylpyruvate; PEP, phosphoenolpyruvate; PP pathway, pentose phosphate pathway.

Here, we attempted to enhance pABA production through the co-utilization of glucose and xylose. The pABA biosynthetic pathway releases PYR during its reaction process, using l-glutamine (l-Gln) as an amino donor. Hence, glucose was utilized as the carbon skeleton for pABA, and xylose was included to support l-Gln synthesis and energy generation. (Fig. 1). We evaluated the effects of deleting genes involved in l-Gln-consuming pathways on pABA production and finally explored different strategies for xylose supplementation. Our results demonstrate that pABA production can be controlled by adjusting the initial ratio of glucose to xylose. We secondly investigated the potential of our co-utilization strategy for the biosynthesis of 4-amino-phenylalanine (4APhe).

## MATERIALS AND METHODS

### Strains and plasmid

The strains used in this study are listed in Table 1. We used *E. coli* ATCC31882 as the host strain and *E. coli* NovaBlue competent cells (Novagen, Cambridge, MA, USA) for gene cloning. For polymerase chain reaction (PCR), KOD FX Neo (TOYOBO, Osaka, Japan) was used with DNA primers purchased from Invitrogen Custom DNA Oligos service (Thermo Fisher Scientific, Tokyo, Japan) or DNA Custom Synthesis service (FASMAC, Atsugi, Japan). The plasmids and primers used are listed in Tables S1 and S2. Genes were assembled with the respective plasmids using the NEBuilder HiFi DNA Assembly Master Mix (New England Biolabs, Ipswich, MA, USA). The pTΔ*glsA* plasmid was constructed as follows: the pTΔglsAF gene fragment was amplified via PCR using pTargetF (20) as the template and N20_del_glsA_F and N20_del_glsA_R as the primers. The amplified fragment was self-ligated, and the resultant plasmid was named pTΔglsAF. Upstream and downstream DNA sequences of *glsA* were amplified using *E. coli* MG1655, with Up_del_glsA_F and Up_del_glsA_R, Down_del_glsA_F and Down_del_glsA_R as the respective primer pairs. The resultant fragments were fused using overlap extension PCR with Up_del_glsA_F and Down_del_glsA_R as the primer pair. The amplified donor was cloned into the *EcoR*I/*Sal*I sites of pTΔglsAF, resulting in the production of the pTΔ*glsA* plasmid. Other plasmids of the pTarget series—namely pTΔ*glsB*, pTΔ*glnB*, pTΔ*glnE*, pTΔ*carAB*, and pTΔ*xylAB*—were constructed using the same procedure as for pTΔ*glsA*. The CRISPR-Cas9 two-plasmid system was used for genetic deletions in the engineered strain with the pTarget and pCas plasmids (20).

**TABLE 1:** Strains used in the present study

Strain	Genotype	Reference
Nova Blue	*endA1*, *hsdR17* (r_K_^−^m_K_^+)^, *supE44 thi-I*, gyrA96*, relA1*, *lac*, *recA1/*F [*proAB^+^*, *lacI*qZΔM15, Tn10(Tet R)]	Novagen
ATCC31882	*aroF aroG tyrR pheA pheA*o *tyrA trpE* (derived from *Escherichia coli* K12)	ATCC
GX1	ATCC31882 *ptsHI*::P_A1lacO-1_-*glk-galP* Δ*pykF* Δ*pykA* Δ*pheA* Δ*tyrA* Δ*eda* Δ*ppc* Δ*pck* Δ*ppsA*	(18)
CFT037	ATCC31882 *ptsHI*::P_A1lacO-1_-*glk-galP* Δ*pykF* Δ*pykA* Δ*eda* Δ*ppc* Δ*pck* Δ*ppsA*Δ*edd*Δ*iclR*Δ*sdaA*	(17)
GX11	GX1Δ*glsA*Δ*glsB*	This study
GX12	GX1Δ*glnB*Δ*glnE*	This study
GX13	GX1Δ*carAB*	This study
GX14	GX1Δ*glsA*Δ*glsB*Δ*carAB*	This study
GX15	GX1Δ*glsA*Δ*glsB*Δ*glnB*Δ*glnE*Δ*carAB*	This study
GX16	CFT037Δ*sdaB*Δ*tdcG*Δ*xylAB*Δ*pheA* Δ*tyrA*	This study
GX17	GX16Δ*nhoA*	This study
GX1B	GX1 harboring pZE12-*pabABC*	This study
GX1BD	GX1B harboring pSAK-D2 plasmid	This study
GX1BW	GX1B harboring pSAK-W plasmid	This study
GX11BW	GX11 harboring pZE12-*pabABC* and pSAK-W plasmids	This study
GX12BW	GX12 harboring pZE12-*pabABC* and pSAK-W plasmids	This study
GX13BW	GX13 harboring pZE12-*pabABC* and pSAK-W plasmids	This study
GX14BW	GX14 harboring pZE12-*pabABC* and pSAK-W plasmids	This study
GX15BW	GX15 harboring pZE12-*pabABC* and pSAK-W plasmids	This study
GX16BW	GX16 harboring pZE12-*pabABC* and pSAK-W plasmids	This study
GX17BW	GX17 harboring pZE12-*pabABC* and pSAK-W plasmids	This study
GX16AW	GX16 harboring pNE-Ptrc-EcpabAB-SvpapBC and pSAK-W plasmids	This study

### Media

Lysogeny broth (LB) medium comprised the following (per liter): 10 g tryptone, 5 g yeast extract, and 5 g NaCl. The recipe for M9Y medium used was: glucose and xylose at various appropriate concentrations, 5 g/L of yeast extract, 0.1 mM CaCl_2_･2H_2_O, 0.01 mM FeSO_4_･7H_2_O, 1 mM MgSO_4_･7H_2_O, 10 mg/L of thiamine hydrochloride, 0.5 g/L of NaCl, 6.7 g/L of Na_2_HPO_4_, 3 g/L of KH_2_PO_4_, 1 g/L of NH_4_Cl, and 0.1 mM IPTG, 40 mg/L l-tyrosine, 40 mg/L l-tryptophan, and 100 mg/L l-phenylalanine. Ampicillin and/or chloramphenicol were added to final concentrations of 100 and 30 mg/L, respectively, where appropriate.

Modified M9Y medium [15 g/L glucose and 5 g/L xylose, 10 mM α-ketoglutarate, 5 g/L yeast extract, 100 mg/L l-tyrosine, 100 mg/L l-tryptophan, 100 mg/L l-phenylalanine, 6 g/L Na_2_HPO_4_, 3 g/L KH_2_PO_4_, 0.5 g/L NaCl, 2 g/L (NH_4_)_2_SO_4_, 0.1 mM CaCl_2_･2H_2_O, 0.01 mM FeSO_4_･7H_2_O, 1 mM MgSO_4_･7H_2_O, 10 mg/L of thiamine hydrochloride, 0.1 mM IPTG, 100 mg/L ampicillin, and 30 mg/L chloramphenicol] was used for bioreactor cultivation. When cultivating GX17BW, glucose/MgSO_4_ rich medium (30 g/L glucose and 10 mM MgSO_4_･7H_2_O) was used.

The feeding medium for fed-batch cultivation of pABA was composed of 600 g/L glucose, 200 g/L xylose, 8 g/L MgSO_4_･7H_2_O, 100 mg/L l-tyrosine, 100 mg/L l-tryptophan, 100 mg/L l-phenylalanine, 0.1 mM CaCl_2_･2H_2_O, 0.01 mM FeSO_4_･7H_2_O, and 10 mg/L of thiamine hydrochloride, 1 mM IPTG, 100 mg/L ampicillin, and 30 mg/L chloramphenicol. When cultivating GX17BW, the feeding medium was as follows: 100 g/L xylose and 200 mg/L ampicillin.

The feed solution for 4APhe production was as follows: 500 g/L glucose, 250 g/L xylose, 8 g/L MgSO_4_･7H_2_O, 100 mg/L l-tyrosine, 100 mg/L l-tryptophan, 100 mg/L l-phenylalanine, 0.1 mM CaCl_2_･2H_2_O, 0.01 mM FeSO_4_･7H_2_O, 10 mg/L of thiamine hydrochloride, 1 mM IPTG, 200 mg/L ampicillin, and 30 mg/L chloramphenicol.

### Culture conditions

Engineered strains were inoculated into 4 mL of M9Y medium in a 15 mL test tube and incubated overnight at 37°C with shaking at 220 rpm. For microbial production, this pre-culture was transferred into a 15 mL test tube containing 5 mL of M9Y medium at an initial optical density (OD)_600_ of 0.1. The test tubes were incubated at 37°C with shaking at 220 rpm.

In the case of bioreactor cultivation, engineered strains were inoculated in 50 mL of M9Y medium containing 20 g/L glucose and 20 g/L xylose and incubated overnight at 37°C with shaking at 220 rpm in a 200 mL flask. Pre-cultures were transferred into a 1 L bioreactor containing 600 mL of modified M9Y medium until the OD_600_ reached 0.1. The temperature and pH were maintained at 30°C and 7.00. The pH was controlled by automated addition of 28% (vol/vol) NH_3_ and 1 N H_2_SO_4._ PLONON 201 (NOF CORPORATION, Tokyo, Japan) was included to prevent foam formation. Dissolved oxygen (DO) was maintained at 30% by controlling agitation. For the cultivation of the GX16BW strain and GX16AW strain (Fig. 6A through D), the feeding solution was added at the rate of 1 mL/min when the DO level increased over 31.2%, and the feeding was automatically stopped when the DO decreased back to 30%. For the cultivation of the GX17BW strain (Fig. 6E through H), the feeding solution (10 mL) was manually added after 47, 71, and 96 h.

### Analytical methods

Cell growth was determined by measuring the OD_600_ using a UVmini-1240 spectrophotometer (Shimadzu Corporation, Kyoto, Japan). Sugars (glucose and xylose) were analyzed using a Prominence HPLC System (Shimadzu) equipped with a Shodex SUGAR KS-801 column (grain diameter: 6 µm, *L* × I.D.: 300 × 8.0 mm^2^; Resonac Holdings Corporation, Tokyo, Japan). The mobile phase was water, run at a rate of 0.8 mL/min at 60°C. The high-performance liquid chromatography (HPLC) profile was detected using a refractive index detector.

We used an HPLC (Shimadzu Corporation) equipped with an MSII column (5 µm, 4.6 mm, I.D. × 250 mm L) (Nacalai Tesque, Kyoto, Japan) for pABA analysis and obtained elution profiles using a 254 nm UV-VIS detector. The two-component system was used with a mobile phase of 0.2% phosphate buffer (A) and methanol (B). The gradient was started with a 70:30 mixture of A and B, shifting to a 50:50 mixture over 2 min beginning at 4 min. This ratio was retained from 6 to 14 min, then returned to the 70:30 ratio by 16 min (over the course of 2 min). The flow rate of the mobile phase was 1.0 mL/min, and the column remained at 40°C.

Samples for 4APhe analysis were labeled using the DL-Amino Acid Labeling Kit (Nacalai Tesque), according to the manufacturer’s instructions. Each sample was analyzed using an HPLC (Shimadzu Corporation) equipped with an MSII column (5 µm, 4.6 mm I.D. × 250 mm L, Nacalai Tesque), and profiles were obtained using a 340 nm UV-VIS detector. The two-component system was used with a mobile phase of 0.2% phosphate buffer (A) and methanol (B). The ratio of the two mobile phase solutions was kept at 50:50. The flow rate of the mobile phase was 1.0 mL/min for 20 min, and the column remained at 40°C.

## RESULTS AND DISCUSSION

### Construction of pABA-producing strain using glucose and xylose as co-substrate

*E. coli* strain GX1 (18), which is derived from ATCC31882 (a phenylalanine overproducer), was used as a host strain for pABA production (Fig. 1). This strain lacks the *pykF*, *pykA*, *eda*, *ppc*, *pck*, and *ppsA* genes and is suitable for the synthesis of shikimate pathway derivatives. The phosphotransferase system was previously replaced with the galactose permease system to block conversion of PEP to PYR. Consequently, the metabolic pathway in the GX1 strain was successfully engineered to promote PEP accumulation. The Dahms pathway converts xylose into glyoxylate and PYR, and so was introduced to restore the PYR supply. This strain was then able to utilize glucose to produce only shikimate derivative, and xylose was used solely for cell growth. This strain was applicable for pABA production as pABA is synthesized from chorismate.

pABA can be also produced in bacteria and yeasts via the shikimate pathway. PEP is produced in glycolysis, and erythrose 4-phosphate is formed in the pentose phosphate pathway, and the condensation of the compounds produces 3-deoxy-d-arabino-heptulosonate 7-phosphate (DAHP). The DAHP is subsequently converted to chorismate. The biosynthesis of pABA from chorismate involves two conversion steps, which are catalyzed by three enzymes via the intermediate 4-amino-4-deoxychorismate (4ADC). In the first step, ADC synthase component II and ADC synthase component I, which are encoded by *pabA* and *pabB*, respectively, substitute the hydroxyl group at position 4 of chorismate with an amino group using glutamine. In the second step, ADC lyase (encoded by *pabC*) eliminates PYR, forming pABA (21, 22).

**Fig 2:**
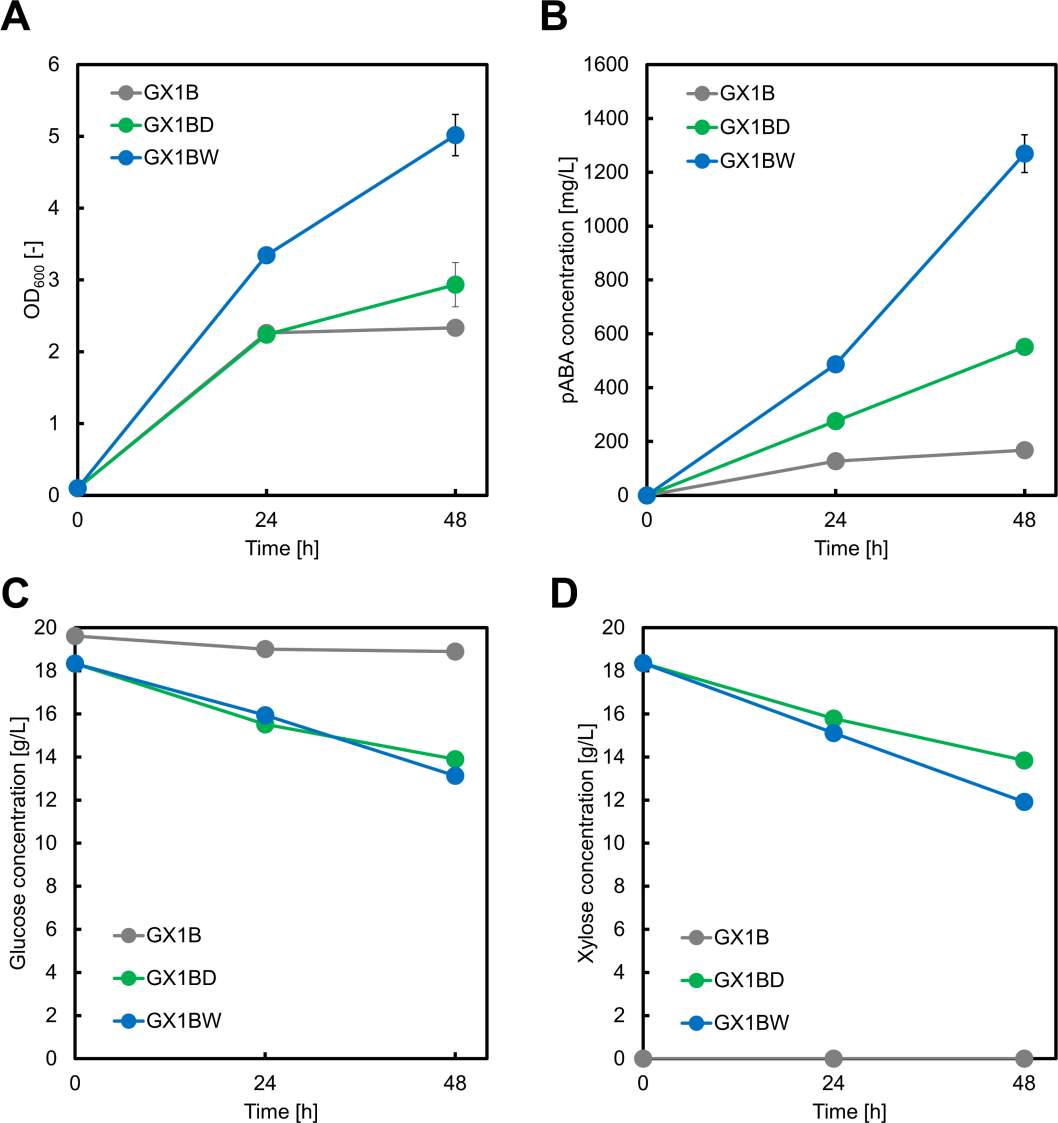
Culture profiles of the GX1B, GX1BD, and GX1BW strains. Each strain included the pZE12-*pabABC* plasmid and either pSAK-D2 or pSAK-W plasmids and were cultivated in M9Y medium. The GX1B strain was grown in 20 g/L glucose, while the GX1BD and GX1BW strains were cultivated in a medium containing 20 g/L each of glucose and xylose. The graphs illustrate (A) cell growth, (B) *p*-aminobenzoic acid concentration, (C) glucose concentration, and (D) xylose concentration. Data are presented as means, and error bars indicate the standard deviation from three independent experiments.

We used the pZE12-*pabABC* (23) plasmid—which contains the *pabA*, *pabB*, and *pabC* genes—to produce pABA from glucose. PYR is released in the final step catalyzed by the product of *pabC*; thus, the GX1B strain (GX1 harboring pZE12-*pabABC*) was anticipated to use glucose as its sole carbon source, as in salicylate synthesis (17). However, we did not observe significant growth when glucose was provided as the sole carbon source (Fig. 2A), and the amount of pABA produced was less than 0.2 g/L (Fig. 2B). To promote the TCA cycle, we introduced the Dahms pathway from *Caulobacter crescentus* as this involves the conversion of xylose to d-xylonolactone by xylose dehydrogenase (XDH), enabling the use of xylose as a carbon source. After nonenzymatic transformation or the reaction catalyzed by xylonolactonase of d-xylonolactone to d-xylonate, endogenous xylonate dehydratases (YjhG) of *E. coli* convert d-xylonate to 2-keto-3-deoxy-d-xylonate. Endogenous aldolases (YjhH) catalyze the conversion of this chemical to glycoaldehyde and PYR. Glycoaldehyde is then metabolized as glycolate through endogenous metabolic activity in *E. coli*, and glycolate is subsequently converted to malate by glyoxylate shunt (Fig. 1) (24).

We introduced the pSAK-D2 plasmid containing *xylB* and *xylC* from *C. crescentus*, *yjhHG* from *E. coli* (19) into the GX1B strain, resulting in the GX1BD strain. After 48 h of cultivation in a medium containing glucose/xylose as co-substrates, 0.55 ± 0.01 g/L pABA was produced (Fig. 2B). However, both GX1B and GX1BD strains showed poor growth (Fig. 2A), with GX1BD consuming 4.44 g/L of glucose (Fig. 2C). In the pABA biosynthetic pathway, α-ketoglutarate (α-KG) is an intermediate in the synthesis of the amino donor l-Gln. To address this limitation, we considered the Weimberg pathway as an alternative to the Dahms pathway. In the Weimberg pathway, XDH converts d-xylose to d-xylonolactone, which is then converted to α-KG via d-xylonate, 2-keto-3-deoxy-d-xylonate, and 2-oxoglutarate semialdehyde. We then introduced pSAK-W (19) into GX1B, generating the GX1BW strain, which produced 1.27 ± 0.07 g/L pABA in the same culture conditions (Fig. 2B), significantly higher than GX1BD. Additionally, cell growth was markedly improved, with GX1BW consuming 5.21 g/L of glucose and 6.44 g/L of xylose (Fig. 2A, C and D). The yield of pABA in GX1BW was 0.24 g/g-glucose; a twofold increase compared to our previous report (0.12 g/g-glucose [11]). These results indicate that manipulating metabolic pathways related to l-Gln synthesis impacts pABA production.

### Deletions of genes for improving l-Gln availability

**Fig 3:**
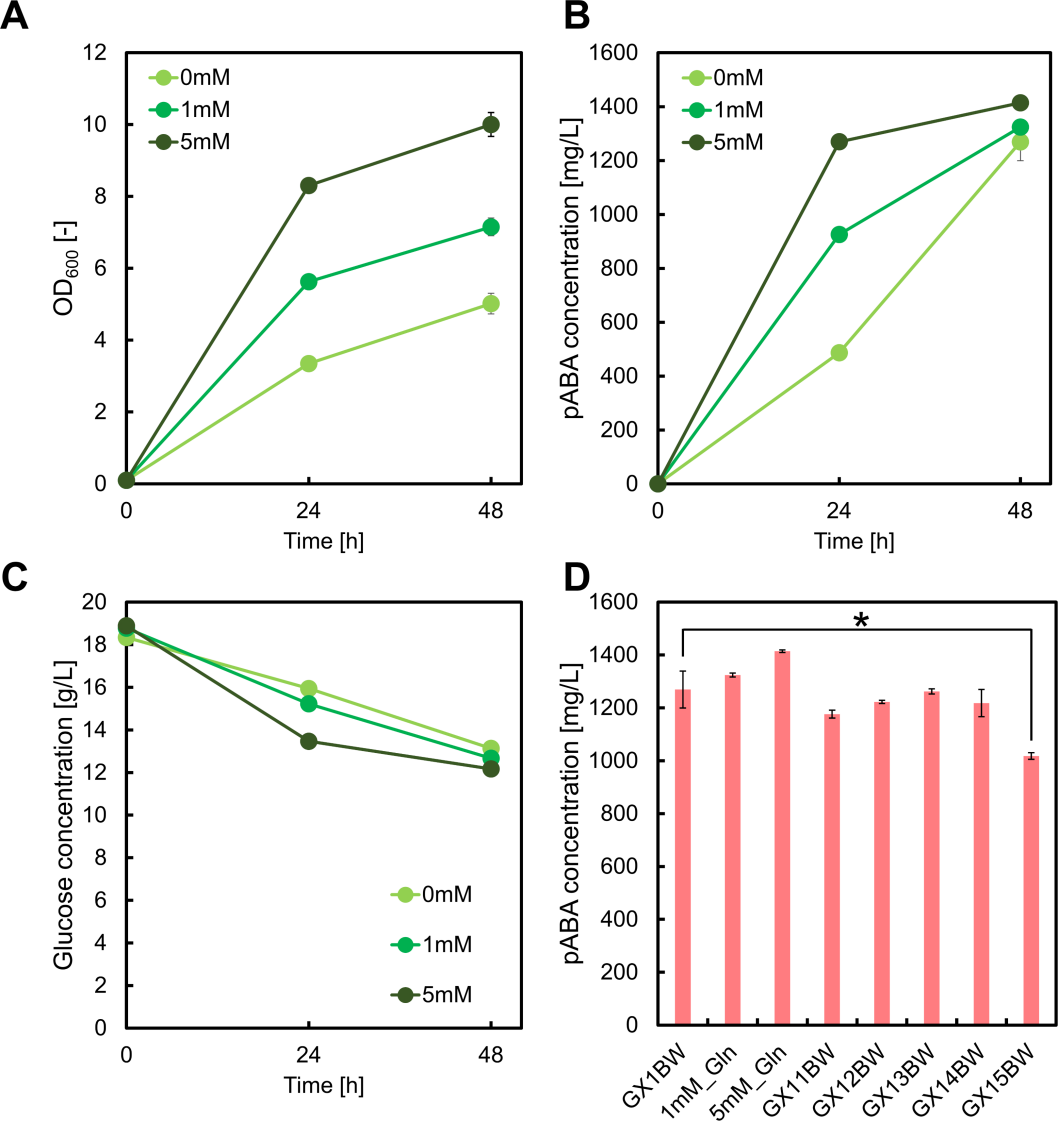
Culture profiles of GX1BW strains. The cell strains contained the pSAK-W and pZE12-*pabABC* plasmids and were cultured in M9Y medium containing glucose and xylose with the addition of l-glutamine. Graphs illustrate (A) cell growth, (B) *para*-aminobenzoic acid concentration, (C) glucose concentration, and (D) pABA titers from GX11BW-GX15BW strains with the pSAK-W and pZE12-*pabABC* plasmids grown in M9Y medium containing 20 g/L each of glucose and xylose. Data are presented as means, and error bars indicate the standard deviation from three independent experiments. *P < 0.05, compared to the GX1BW strain.

We evaluated the effect of l-Gln on pABA production in GX1BW. Cultivation of GX1BW in M9Y broth containing 20 g/L each of glucose and xylose, supplemented with 1 mM or 5 mM l-Gln, revealed that l-Gln improved cell growth and sugar consumption rate (Fig. 3A and C). After 48 h of cultivation, the titer of pABA was slightly increased with the 5 mM of l-Gln supplement (Fig. 3B), indicating that l-Gln replenishes the slight depletion of substrates around the TCA cycle and improving the supply of l-Gln in GX1BW can increase pABA titer.

To improve l-Gln availability for pABA synthesis, we disrupted three specific pathways in the GX1 strain: *glsAB*, *glnBE*, and *carAB*. The *glsA* and *glsB* genes encode glutaminases (GlsA and GlsB), which catalyze the reverse reaction of l-Gln synthesis. The *glnB* and *glnE* genes encode PII-1 protein and glutamine synthetase adenylyltransferase/deadenylase, respectively, which diminish glutamine synthetase (encoded by *glnA*) activity involved in l-Gln production. Finally, *carA* and *carB* encode carbamoyl-phosphate synthase subunits involved in the synthesis of citrulline and arginine. A previous study demonstrated that the deletion of *glnB* and *glnE* improves l-Gln production by 24.1% (25). Knockout of the *glsA*, *glsB*, *glnB*, *glnE*, and *lpxM* genes resulted in the production of more than twice as much l-Gln compared to the control. We constructed the GX1Δ*glsAB*, GX1Δ*glnBE*, and GX1Δ*carAB* strains and introduced both pZE12-*pabABC* and pSAK*-*W plasmids into these strains, resulting in GX11BW, GX12BW, and GX13BW strains. After 48 h of cultivation, the GX11BW, GX12BW, and GX13BW strains produced 1.18 ± 0.01, 1.22 ± 0.01, and 1.26 ± 0.01 g/L pABA, respectively (Fig. 3D). Disruption of *glsAB* and *carAB* in GX1 resulted in the GX14 strain. GX14BW harboring pZE12-*pabABC* and pSAK*-*W plasmids produced 1.22 ± 0.05 g/L pABA. Additional disruption of *glnB* and *glnE* in GX14, resulting in the GX15BW strain, produced 1.02 ± 0.01 g/L pABA from glucose. Combinatorial disruption of the three pathways did not improve pABA titer from glucose. These results indicate that improving the efficacy of l-Gln utilization is not effective for increasing pABA production.

### Control of pABA production by optimization of glucose/xylose ratio and eliminating carbon leakage

**Fig 4:**
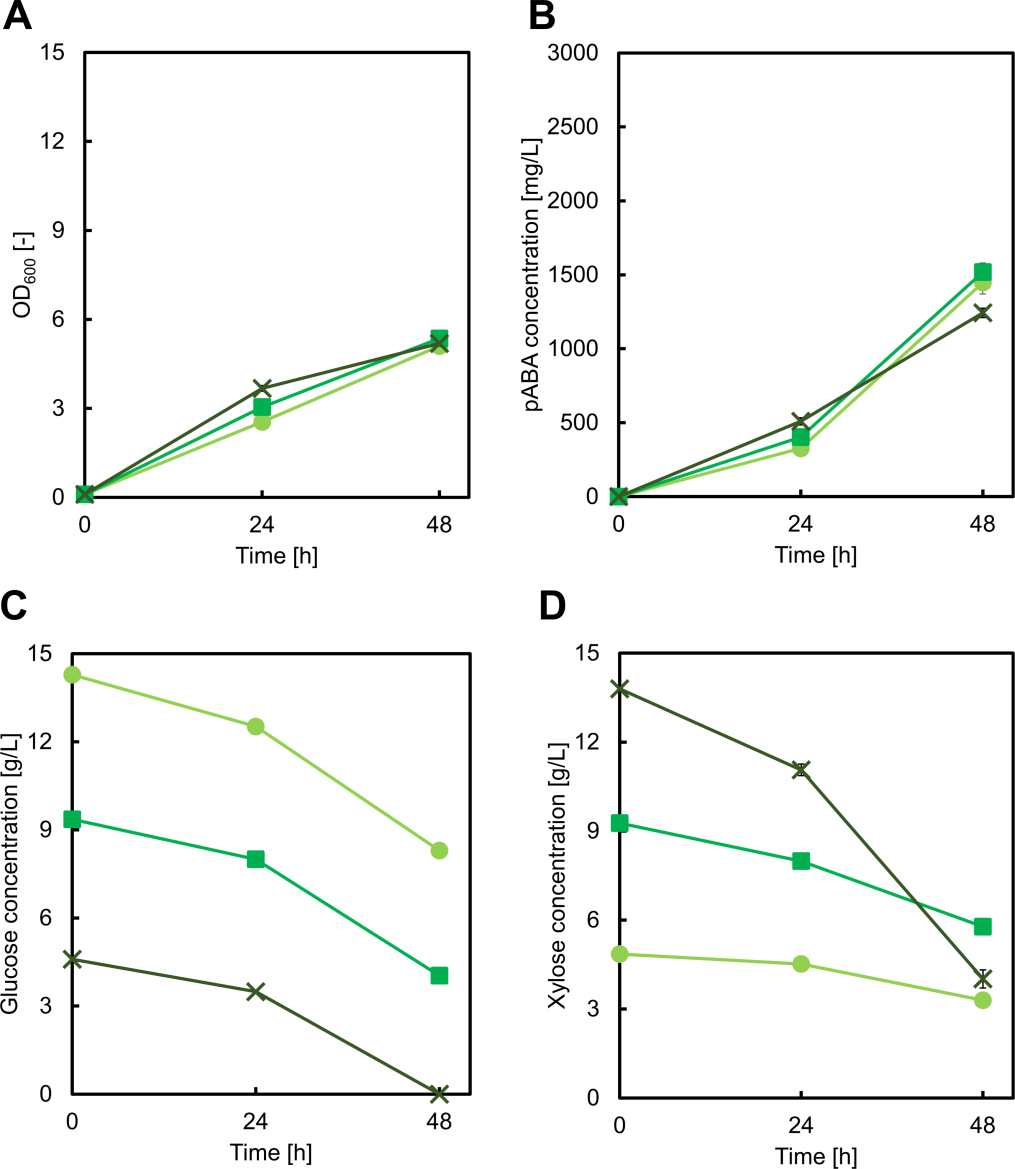
Culture profiles of the GX1BW strain cultured in M9Y medium containing 15 g/L glucose and 5 g/L xylose (light green circles), 10 g/L glucose and 10 g/L xylose (green squares), or 5 g/L glucose and 15 g/L xylose (dark green crosses). Graphs illustrate (A) cell growth, (B) *para*-aminobenzoic acid concentration, (C) glucose concentration, and (D) xylose concentration. Data are presented as means, and error bars indicate the standard deviation from three independent experiments.

Optimizing the ratio of supplied glucose to xylose was a potential avenue for further improvement of pABA production. For this, we cultivated the GX1BW strain in M9Y medium containing both glucose and xylose as follows: 15 g/L glucose and 5 g/L xylose, 10 g/L each glucose and xylose, and 5 g/L glucose and 15 g/L xylose. There was no significant difference in pABA production, cell growth, or glucose uptake in these conditions (Fig. 4A through C). However, the higher initial concentration of xylose led to more rapid xylose uptake (Fig. 4D), suggesting that the endogenous xylose isomerase pathway in *E. coli* might have been activated.

Due to the complexity of the PEP and PYR-related pathways, controlling these pathways represents a key challenge for shikimate-pathway-derived compound biosynthesis. One effective approach to overcome this challenge is the inactivation of serine deamination mediated by l-serine deaminase (LSD). There are three isozymes of LSD in *E. coli*: LSD1, LSD2, and LSD3, encoded by *sdaA*, *sdaB*, and *tdcG*, respectively. While *sdaA* is active under anaerobic conditions in rich media (26), *sdaB* and *tdcG* are activated in glucose-limited conditions (27, 28). Interestingly, all three LSDs are active under aerobic, glucose-rich conditions during maleate production (17).

**Fig 5:**
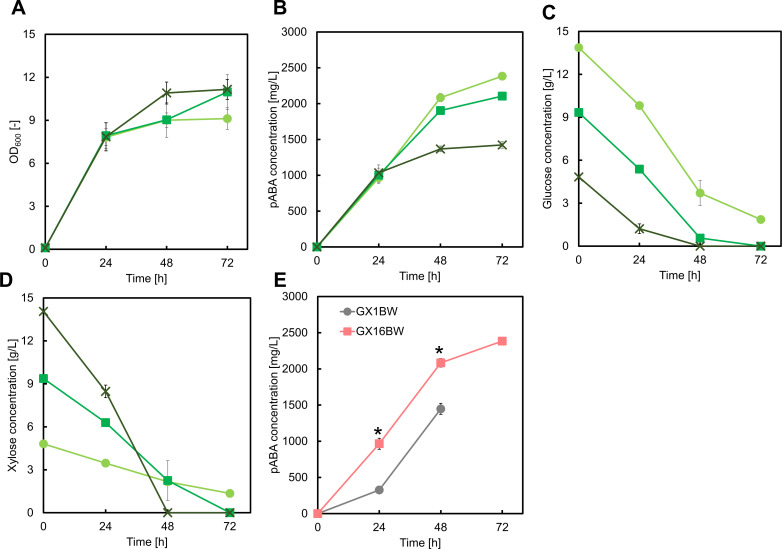
Culture profiles of the GX16BW strain cultured in M9Y medium containing 15 g/L glucose and 5 g/L xylose (light green circles), 10 g/L glucose and 10 g/L xylose (green squares), or 5 g/L glucose and 15 g/L xylose (dark green crosses). Graphs illustrate (A) cell growth, (B) *para*-aminobenzoic acid concentration, (C) glucose concentration, and (D) xylose concentration. (E) The comparison of pABA production between GX1BW and GX16BW strains in M9Y medium containing 15 g/L glucose and 5 g/L xylose as carbon sources. Data are presented as means, and error bars indicate the standard deviation from three independent experiments. *P < 0.05, compared to the GX1BW strain.

To fully decouple the pABA biosynthetic pathway from those involved in cell growth, we disrupted both the xylose isomerase and serine deamination pathways. We constructed a GX16 strain by disrupting the *sdaB*, *tdcG*, and *xylAB* genes in the CFT037 strain (17) and cultivated the GX16BW strain, which contained the pZE12-*pabABC* and pSAK-W plasmids, in M9Y broth containing glucose and xylose. Disrupting the serine deamination and xylose isomerase pathways resulted in increased pABA titer (Fig. 5B). After 72 h of cultivation in M9Y broth containing 15 g/L glucose and 5 g/L xylose, the GX16BW strain produced 2.38 ± 0.04 g/L pABA, with a yield of 0.20 ± 0.01 g/g from glucose. Additionally, the consumption of both glucose and xylose was increased compared to the GX1BW strain (Fig. 5C and D). Increased glucose consumption was directly correlated with higher pABA production, indicating that enhanced glucose utilization contributes to improved pABA titer. This aligns with previous studies on the CFT591 strain, which lacks any LSD enzymes and exhibits improved cell growth and glucose consumption (17). Additionally, increased xylose uptake following disruption of *xylAB* suggests that the GX16BW utilizes xylose through an exogenous xylose assimilation pathway (the Weimberg pathway). This highlights the feasibility of controlling pABA production by regulating xylose consumption to drive the TCA cycle and production of the amino donor.

### Fed-batch cultivation for pABA production using glucose/xylose as co-substrates

**Fig 6:**
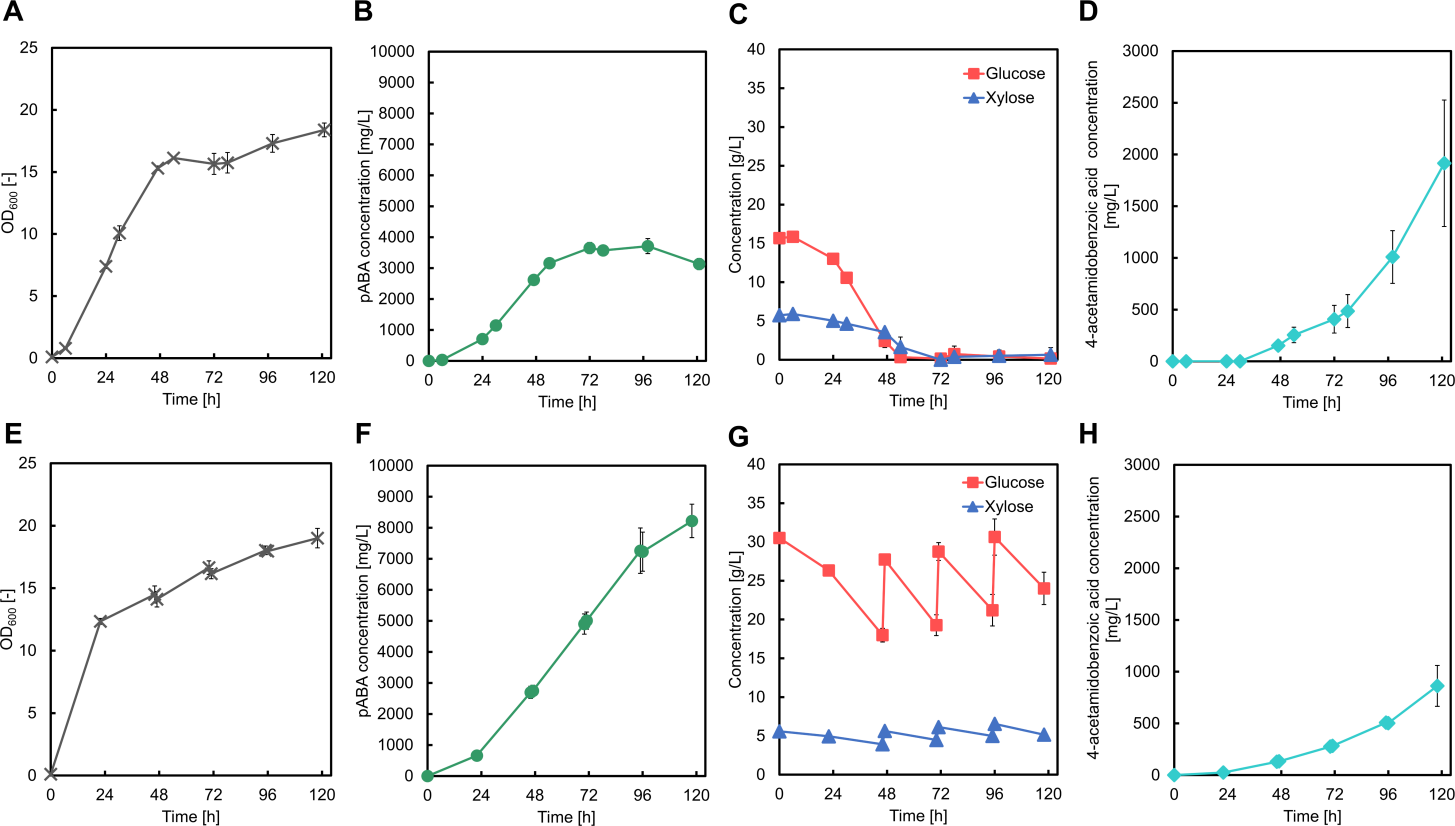
Results of fed-batch cultivation of GX16BW and GX17BW strains for the production of *para*-aminobenzoic acid (pABA). Graphs illustrate the results for GX16BW (A–D) and GX17BW (E–H) for (A and E) cell growth, (B and F) *para*-aminobenzoic acid concentration, (C and G) glucose and xylose concentration, and (D and H) 4-acetamidobenzoic acid concentration. Data are presented as means, and error bars indicate the standard deviation from three independent experiments.

We cultivated the GX16BW strain to further enhance pABA production using fed-batch fermentation in a 1 L bioreactor. After 121 h of cultivation with a glucose-xylose co-substrate, the strain produced 3.13 g/L of pABA (Fig. 6A through C). However, rapid accumulation of 4-acetamidobenzoic acid was observed after 72 h of cultivation (Fig. 6D), which coincided with the cessation of further increase in pABA production (Fig. 6B). By the end of cultivation, 1.91 g/L 4-acetamidobenzoic acid had accumulated (Fig. 6D). Arylamine N-acetyltransferase (encoded by *nhoA*) has been reported to convert pABA to 4-acetamidobenzoic acid by acetylation (29, 30). Considering that this undesirable side reaction might accelerate pABA acetylation during the later stages of cultivation, we disrupted the *nhoA* gene in GX16 to generate GX17. Unexpectedly, the GX17BW strain harboring the pZE12-*pabABC* and pSAK-W plasmids continued to accumulate 4-acetamidobenzoic acid despite the absence of the *nhoA* gene (Fig. S1). This observation suggests that other factors besides *nhoA* expression may contribute to pABA acetylation. For example, nonenzymatic acetylation of lysine residues occurs primarily during the stationary phase and involves acetyl-phosphate (31–33). It is plausible that a similar nonenzymatic mechanism is responsible for pABA acetylation during the stationary phase. Acetyl-CoA availability has been found to improve during sulfur or magnesium starvation when *E. coli* is cultivated with low glucose concentrations (34). Based on this, we cultivated the GX17BW strain in both glucose- and MgSO_4_-rich medium. After 118 h of cultivation, 8.22 g/L pABA was produced (Fig. 6E through G), equivalent to a yield of 0.23 g/g glucose, which is the highest reported level of pABA production in *E. coli*. (Table S3). The accumulation of 4-acetamidobenzoic acid accumulation was also significantly reduced to 0.86 g/L at the end of cultivation (Fig. 6H). This suggests that MgSO_4_ may contribute to pABA acetylation. Further investigation into the mechanisms underlying pABA acetylation could provide valuable insights for developing strains of conditions that eliminate 4-acetamidobenzoic acid accumulation completely, leading to even higher pABA production capabilities.

### Production of 4APhe in pathway-engineered strains

Following the success of our strategy with a PYR-emitting biosynthetic pathway, we sought to test the effectiveness of this approach with a pathway that does not release PYR. We selected 4APhe as a target compound, which is synthesized from chorismate without PYR release, in a pathway that is identical to the pABA synthetic pathway up to 4ADC synthesis (35, 36). Instead, 4-amino-4-deoxy-chorismate mutase (*papB*) and 4-amino-4-deoxyprephenate dehydrogenase (*papC*) convert 4ADC into *p*-aminophenylpyruvate (PAPP), which is subsequently transaminated to yield 4APhe (Fig. 1) (37, 38). We therefore hypothesized that a more direct influence would be observed on 4APhe production compared to the pABA production. Similar to pABA, 4APhe biosynthesis requires an amino donor; thus, we controlled amino donor availability to optimize 4APhe production by adjusting the initial glucose/xylose concentrations.

**Fig 7:**
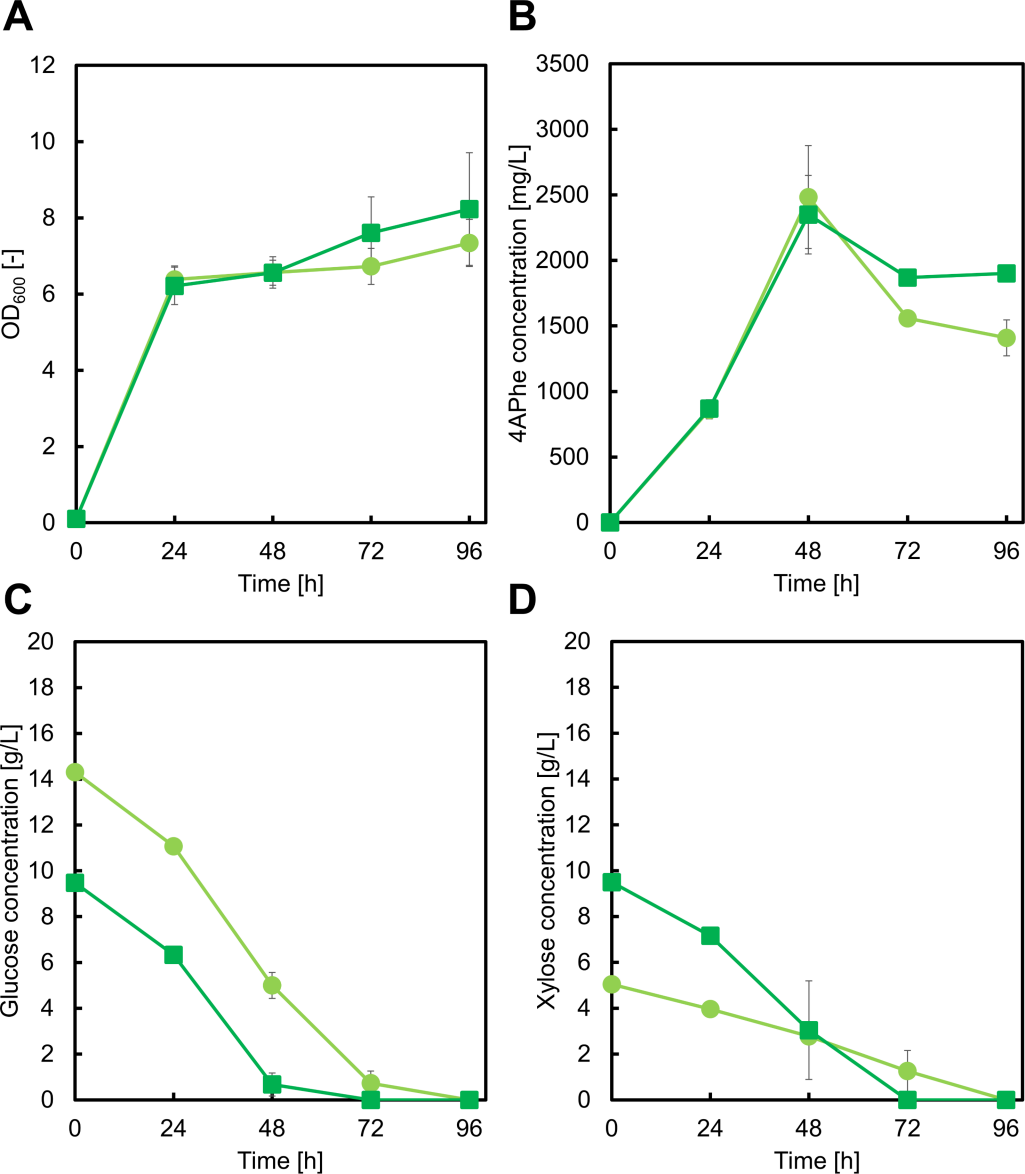
Culture profiles using the GX16AW in M9Y medium containing 15 g/L glucose and 5 g/L xylose (light green circles), 10 g/L each of glucose and xylose (green squares). Graphs illustrate (A) cell growth, (B) 4-amino-phenylalanine (4APhe) concentration, (C) glucose concentration, and (D) xylose concentration. Data are presented as means, and error bars indicate the standard deviation from three independent experiments.

We first constructed the 4APhe-producing strain GX16AW, which harbors the pNE-Ptrc-EcpabAB-SvpapBC (39) and pSAK-W plasmids. We cultivated these strains in a medium containing 15 g/L glucose and 5 g/L xylose or 10 g/L each of glucose and xylose. After 48 h of cultivation in the high-glucose medium, GX16AW produced 2.48 ± 0.39 g/L 4APhe, corresponding to a yield of 0.27 ± 0.05 g/g from glucose (Fig. 7A and B). Although the product yields from glucose of 4APhe and pABA were equivalent, cell growth of the GX16AW strain was slightly lower despite similar sugar utilization (Fig. 7A, C and D). This discrepancy may be attributed to the absence of PYR emission during 4APhe biosynthesis, resulting in reduced carbon flux into the TCA cycle. Notably, when 4APhe concentration declined (after 48 h of cultivation), nearly all the available sugars had been depleted, indicating degradation of 4APhe.

**Fig 8:**
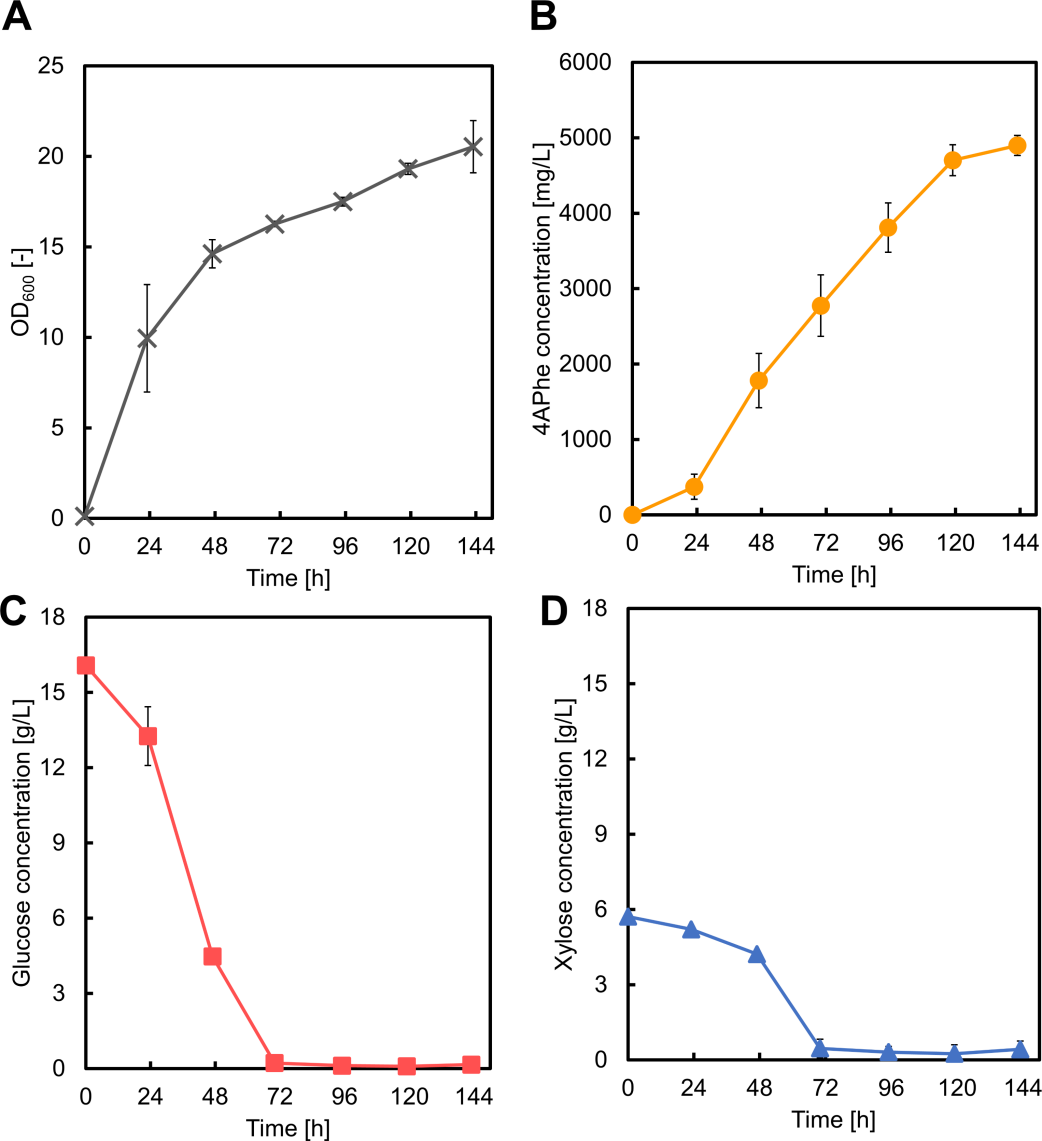
Results of fed-batch cultivation for 4-amino-phenylalanine (4APhe) production using GX16AW. Graphs illustrate (A) cell growth, (B) 4APhe concentration, (C) glucose concentration, and (D) xylose concentration. Data are presented as means, and error bars indicate the standard deviation from three independent experiments.

Fed-batch fermentation with simultaneous utilization of glucose and xylose enabled us to improve 4APhe production, with 4.90 g/L of 4APhe produced after 143 h of cultivation (Fig. 8). We further improved 4APhe production by cultivating the GX16AW strain in MgSO_4_-rich medium, similar to the conditions used for enhanced pABA production. However, no significant increase in 4APhe titer was observed (data not shown). While pABA production benefits from the release of PYR, which supports the energy module, 4APhe biosynthesis does not involve PYR release. Thus, the energy module in 4APhe production is not enhanced by MgSO_4_-rich medium, meaning that further optimization of the 4APhe biosynthetic pathway is needed if the titer of 4APhe is to be improved.

### Conclusions

Our study demonstrates that the co-utilization strategy involving the division of metabolic pathways into distinct production and energy modules is an effective approach for enhancing pABA and 4APhe production in *E. coli*. By optimizing initial sugar concentrations and eliminating pathways responsible for carbon leakage, pABA production is greatly improved to achieve titers of up to 8.22 g/L. To the best of our knowledge, this is the highest pABA production from engineered *E. coli* to date. Furthermore, we achieved high levels of 4APhe production despite the absence of PYR release in the biosynthetic pathway. This provides valuable insights into the development of co-utilization strategies for microbial production of various chemicals, particularly for cases where the biosynthetic pathway requires specific donors such as an amino group. Notably, the co-substrate approach offers a more flexible and rapid means of optimizing biosynthetic pathways compared to extensive genetic modifications. However, we acknowledge that in this study, the impact of substrate ratio on production levels was less pronounced than anticipated. Further investigation is necessary to delineate the relative contributions of co-substrate utilization and metabolic engineering to yield enhancement, as well as to elucidate the broader implications of this approach for microbial chemical biosynthesis.

## Data Availability

The data that support the findings of this study are available from the corresponding author upon reasonable request.

## SUPPLEMENTAL MATERIAL

Supplemental material aem.02468-24-s0001.docx Figures S1 to S8; Tables S1 to S3.

ASM does not own the copyrights to Supplemental Material that may be linked to, or accessed through, an article. The authors have granted ASM a non-exclusive, world-wide license to publish the Supplemental Material files. Please contact the corresponding author directly for reuse.
