# Supplementary material for: Modular pathway engineering for enhanced production of para-aminobenzoic acid and 4-amino-phenylalanine in Escherichia coli via glucose/xylose co-utilization
Source: Appl Environ Microbiol. 2025 Apr 17;91(5):e02468-24. doi: 10.1128/aem.02468-24 (PMC12093962; doi:10.1128/aem.02468-24)
Supplement: Supplemental material — Figures S1 to S8; Tables S1 to S3. [file aem.02468-24-s0001.docx]

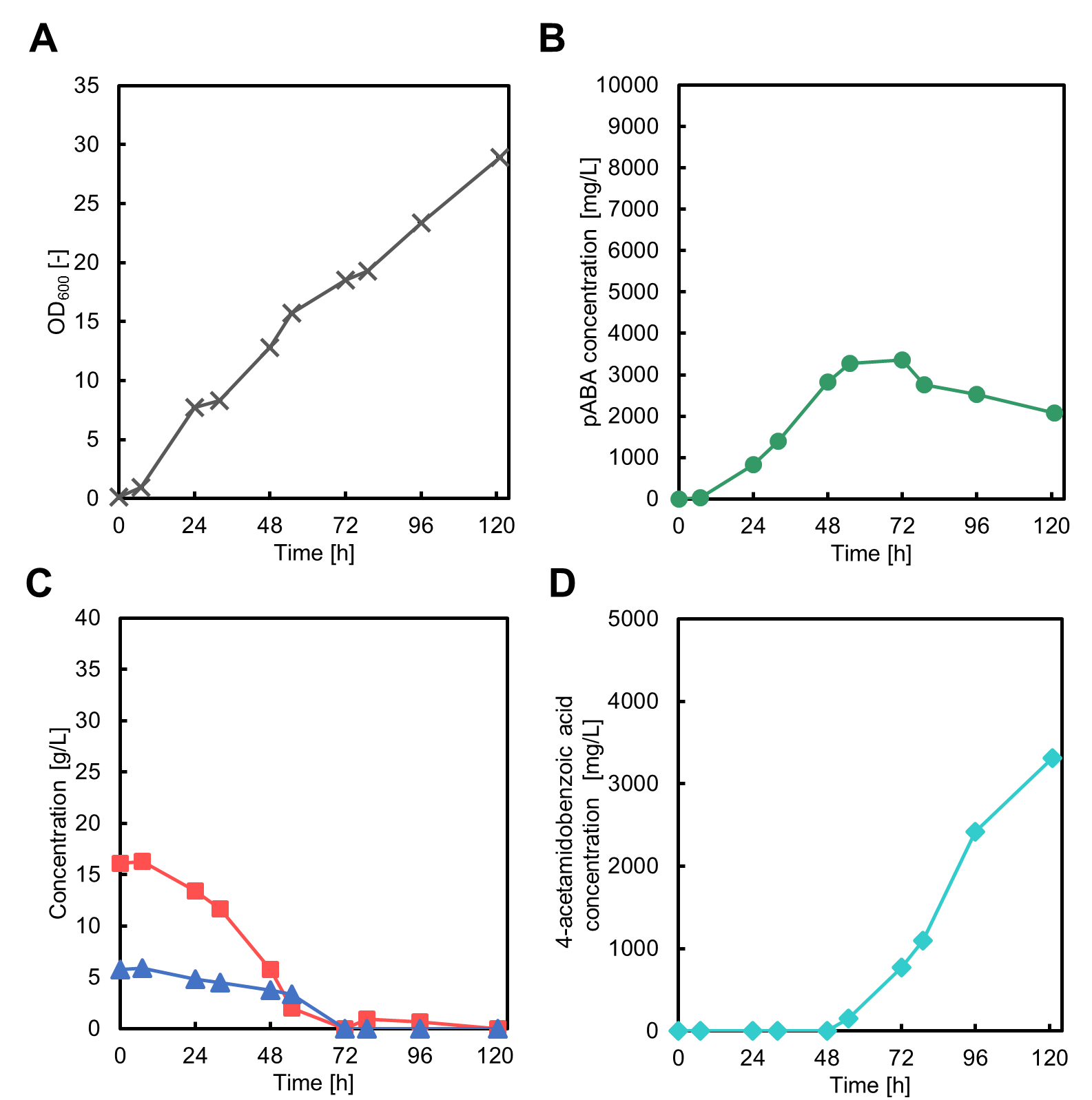


**Supplementary Figure.1**

Fed-batch cultivation for pABA production using the GX17BW (A-D) in modified M9Y medium. Each graph indicated (A) Cell growth (B) pABA concentration (C) concentrations of glucose and xylose (D) 4-acetamidobenzoic acid concentration, respectively. Data shown are n=1.


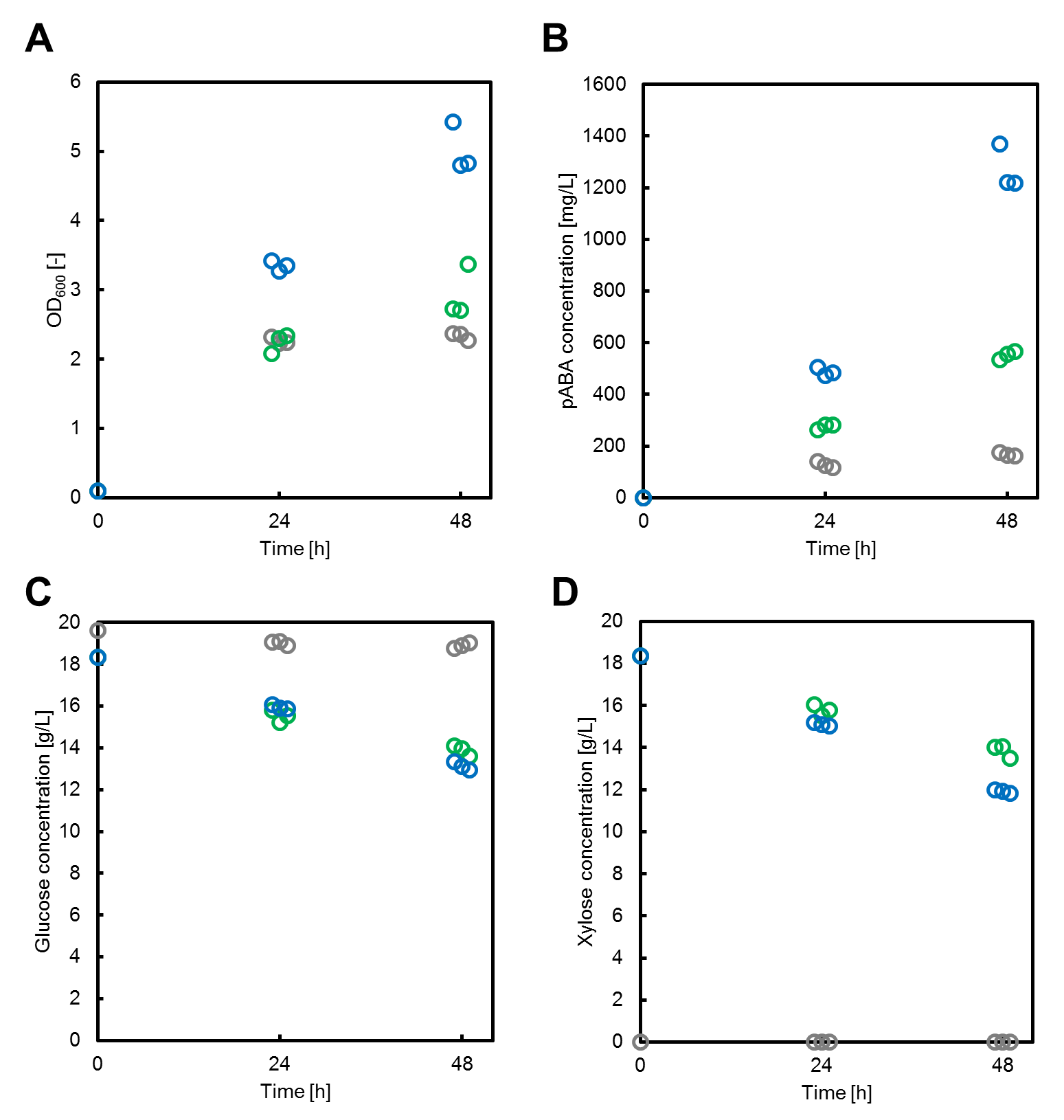


**Supplementary Figure.2** Individual data points of Fig. 2


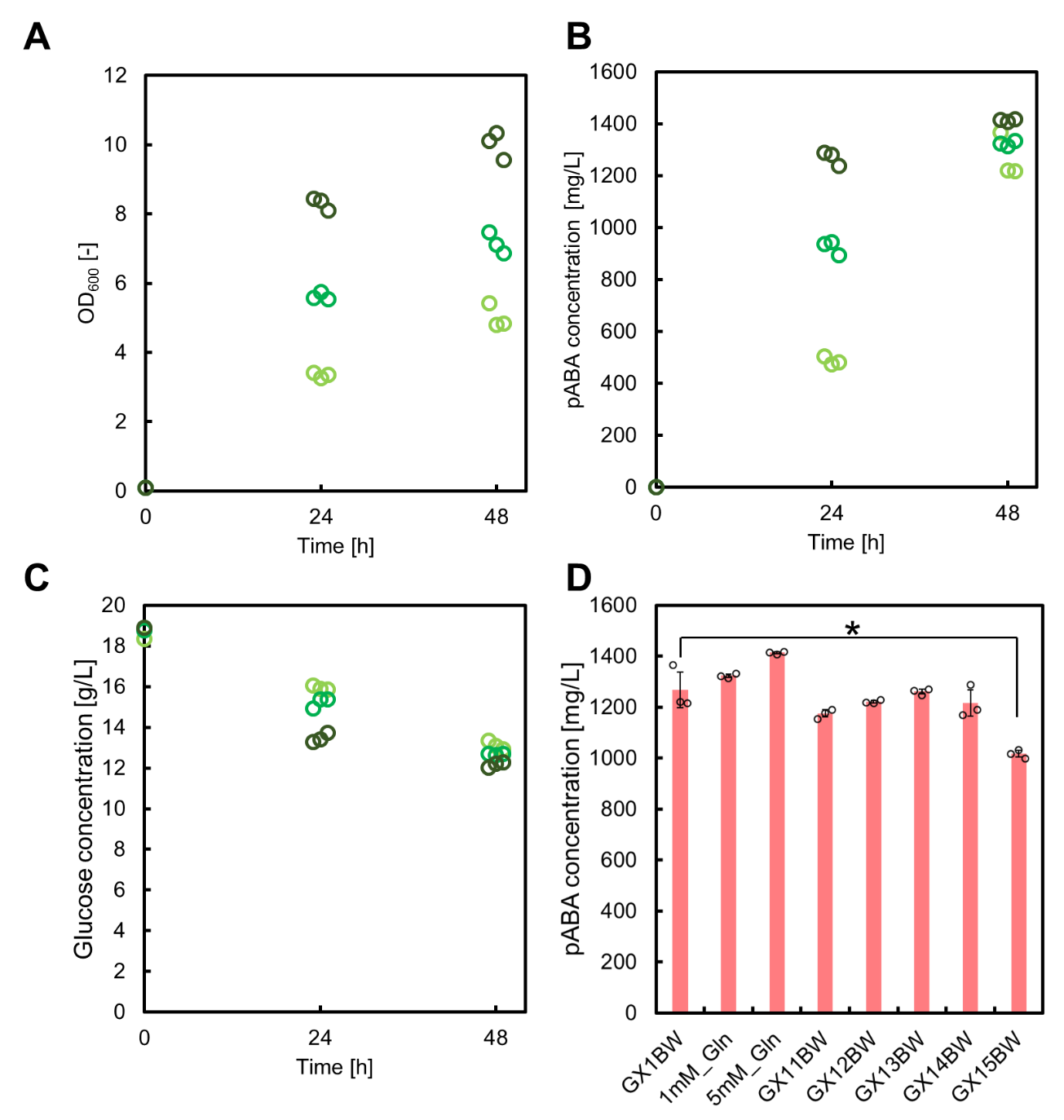


**Supplementary Figure.3** Individual data points of Fig. 3


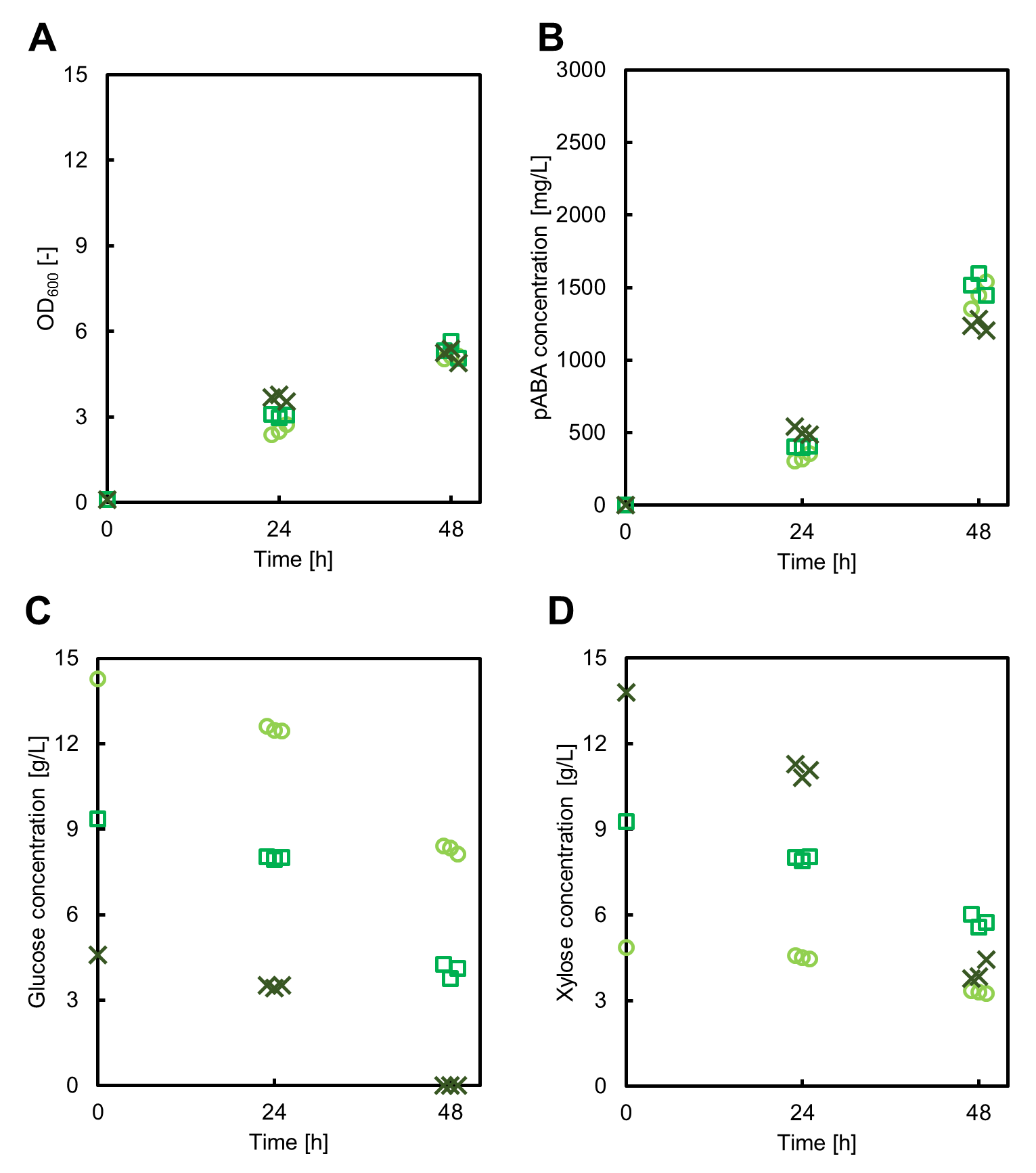


**Supplementary Figure.4** Individual data points of Fig. 4


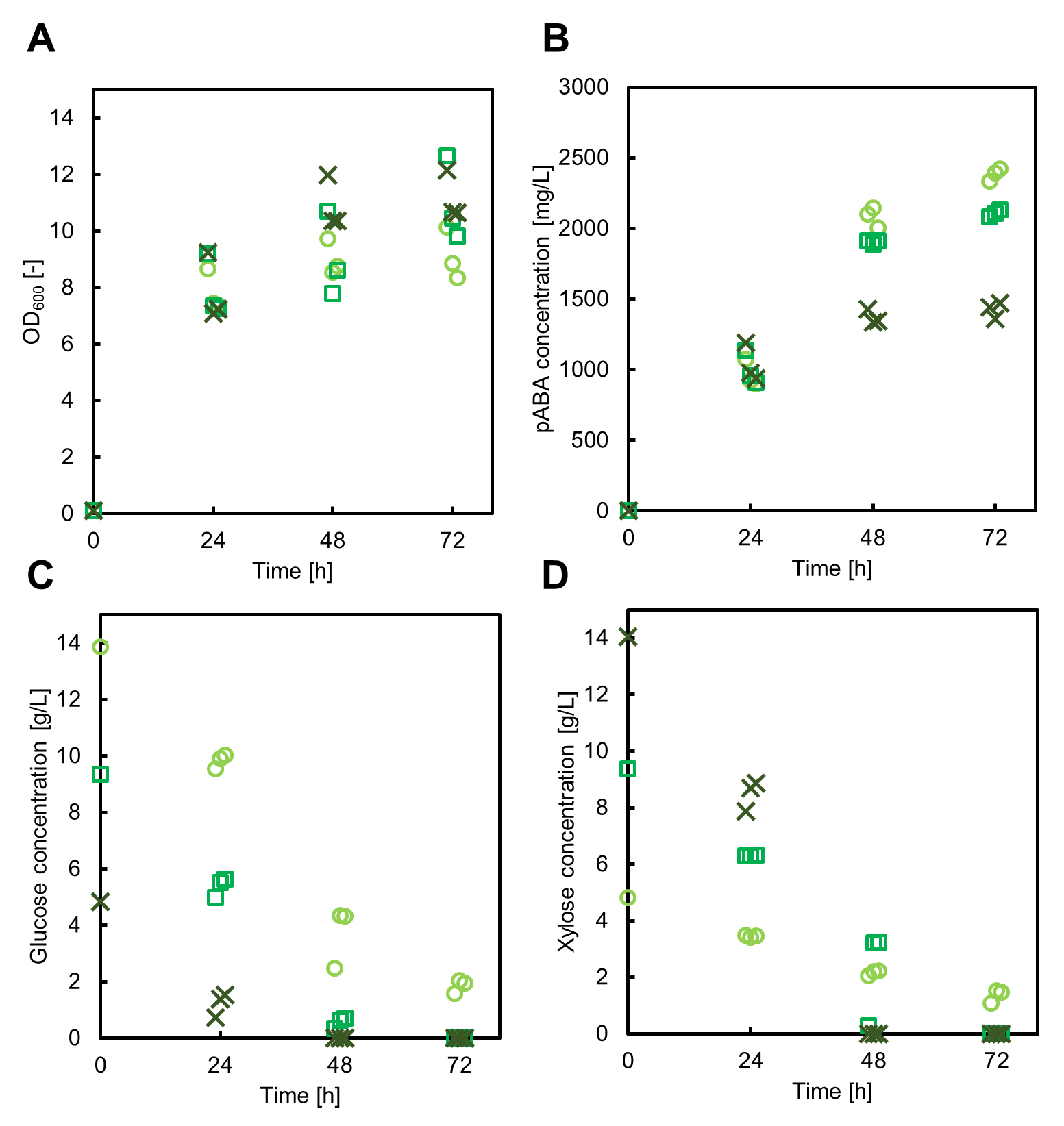


**Supplementary Figure.5** Individual data points of Fig. 5

**
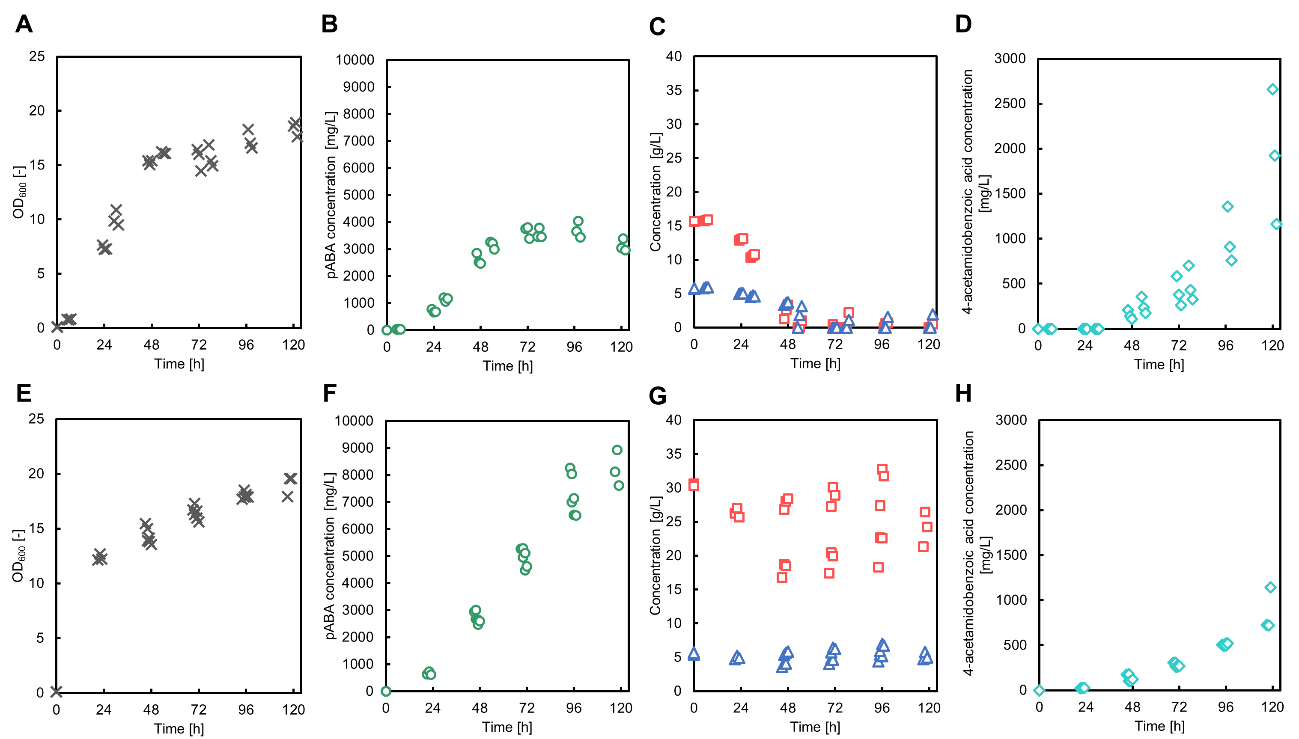
**

**Supplementary Figure.6** Individual data points of Fig. 6

**
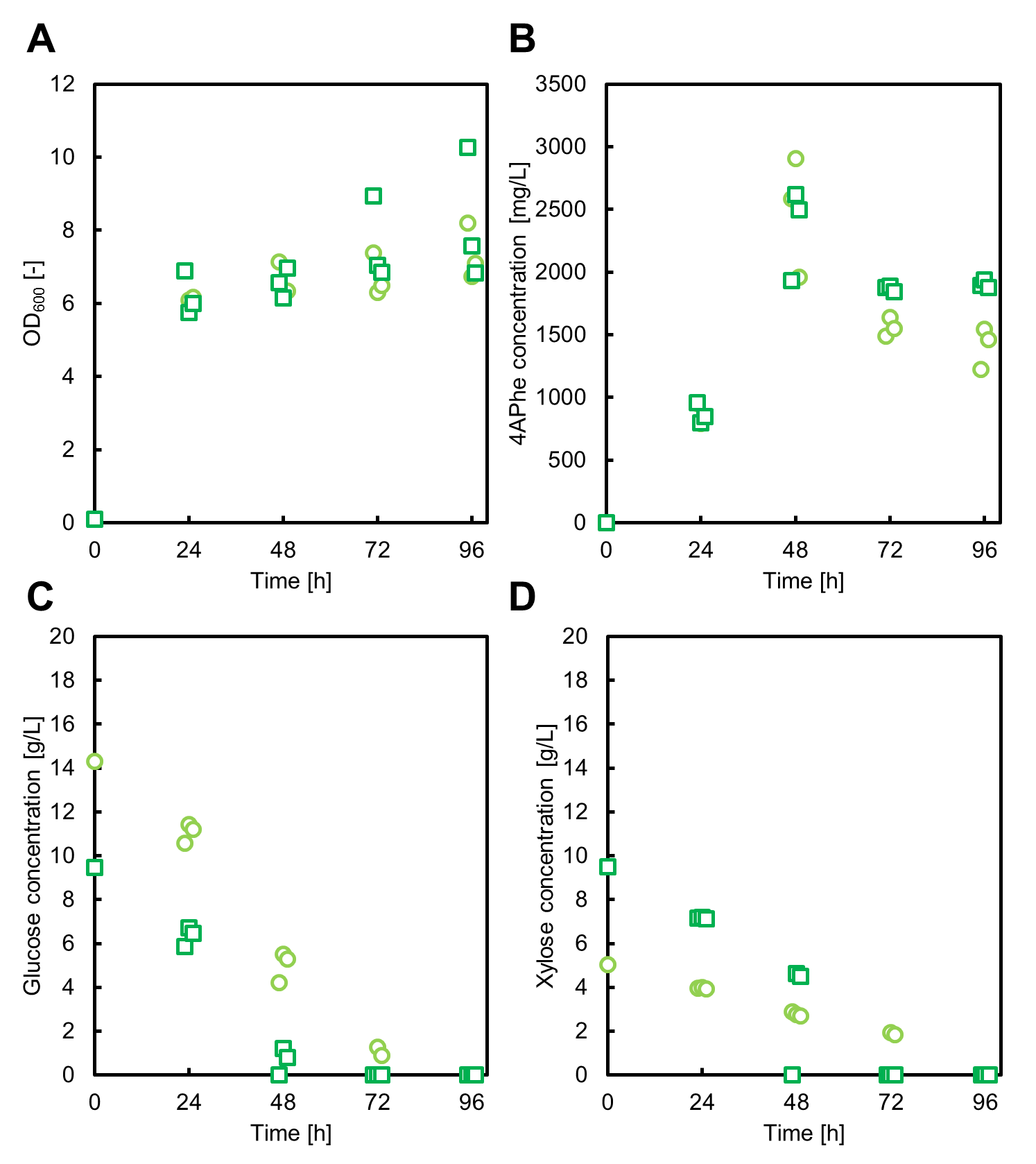
**

**Supplementary Figure.7** Individual data points of Fig. 7

**
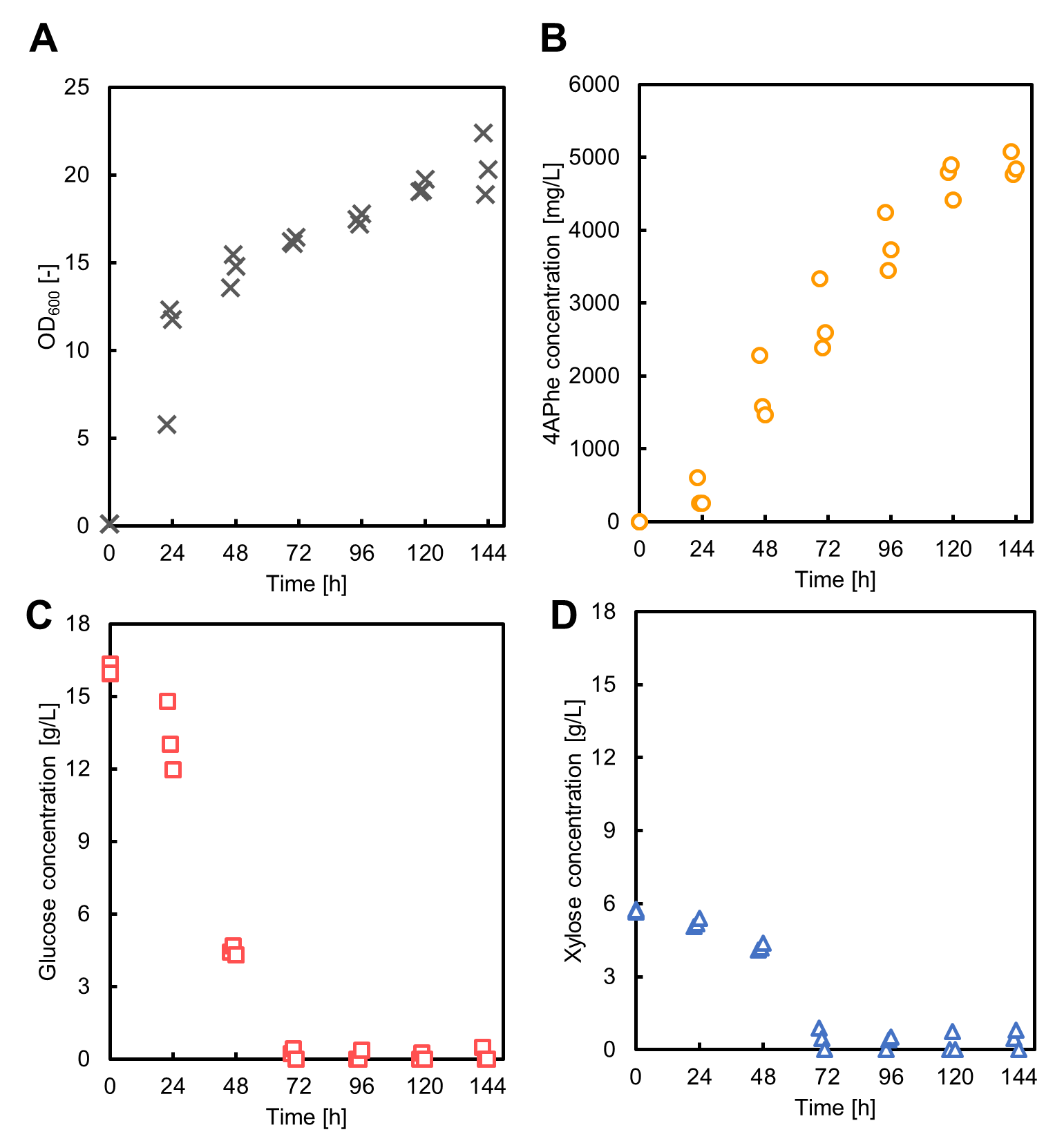
**

**Supplementary Figure.8** Individual data points of Fig. 8

Table S1. Plasmids used in this study

| Plasmids | Characteristics | Reference |
| --- | --- | --- |
| pZE12MCS | P_LlacO-1_, colE *ori*, Amp^r^ | Expressys |
| pSAK | P_A1lacO-1_, pSC101 *ori*, Cm^r^ | [18] |
| pTrcHis B | P_trc_, pBR322 ori, Amp^r^ | Life Technologies |
| pNE12-Ptrc | P_trc_, colE1 ori, Amp^r^ | [39] |
| pZE12-*pabABC* | pZE12MCS containing *pabA*, *pabB* and *pabC* from *E.coli* | [23] |
| pNE-Ptrc-*EcpabAB*-*SvpapBC* | pNE12-Ptrc containing *pabA* and *pabB* from *E. coli* and *svpapB* and *svpapC* from *S. venezuelae* | [39] |
| pSAK-D2 | pSAK containing *xylB* from *C. crescentus* (codon-optimized for *E. coli*), *xylC* from *C. crescentus* (codon-optimized for *E.coli*), and *yjhHG* from *E. coli* | [19] |
| pSAK-W | pSAK containing *xylA*, *xylX* and *xylB* from *C.crescentus* (codon-optimized for *E.coli*) | [19] |
| pTargetF | Constitutive expression of sgRNA | Addgene |
| pCas | Constitutive expression of cas9 and inducible expression of λ RED and sgRNA | Addgene |
| pTΔ*glsA* | Constitutive expression of sgRNA with donor editing template DNA for *glsA* disruption | This study |
| pTΔ*glsB* | Constitutive expression of sgRNA with donor editing template DNA for *glsB* disruption | This study |
| pTΔ*glnB* | Constitutive expression of sgRNA with donor editing template DNA for *glnB* disruption | This study |
| pTΔ*glnE* | Constitutive expression of sgRNA with donor editing template DNA for *glnE* disruption | This study |
| pTΔ*carAB* | Constitutive expression of sgRNA with donor editing template DNA for *carAB* disruption | This study |
| pTΔ*xylAB* | Constitutive expression of sgRNA with donor editing template DNA for *xylAB* disruption | This study |
| pTΔ*nhoA* | Constitutive expression of sgRNA with donor editing template DNA for *nhoA* disruption | This study |
| pTΔ*sdaB* | Constitutive expression of sgRNA with donor editing template DNA for *sdaB* disruption | [17] |
| pTΔ*tdcG* | Constitutive expression of sgRNA with donor editing template DNA for *tdcG* disruption | [17] |
| pTΔ*tyrA* | Constitutive expression of sgRNA with donor editing template DNA for *tyrA* disruption | [18] |
| pTΔ*pheA* | Constitutive expression of sgRNA with donor editing template DNA for *pheA* disruption | [18] |

Table S2. Primers used in this study

| Primer names | Sequence |
| --- | --- |
| N20_del_glsA_F | ATTTCACTCACTTAACGGGGTTTTAGAGCTAGAAATAGCAAGTTAAAATAAGGC |
| N20_del_glsA_R | GTTAAGTGAGTGAAATTGGCTAGCATTATACCTAGGACTGAGCTAGCTGTCAAGG |
| Up_del_glsA_F | TGCTTTTTTTGAATTCCCGCCAGGTTTACCCGTGCCTGAG |
| Up_del_glsA_R | CTACCTGCTCGCCAGCGATGCGGGAGGTAATTCCTCACCCCG |
| Down_del_glsA_F | CCTCCCGCATCGCTGGCGAGCAGGTAGCGCTCTCTG |
| Down_del_glsA_R | GCTTCTGCAGGTCGACCATAGGCATAACCGGAAAACATCGCCACAATAC |
| N20_del_glsB_F | GGGCGATTAAGCGCACCAGGTTTTAGAGCTAGAAATAGCAAGTTAAAATAAGGC |
| N20_del_glsB_R | GTGCGCTTAATCGCCCTTGCTAGCATTATACCTAGGACTGAGCTAGCTGTCAAGG |
| Up_del_glsB_F | TGCTTTTTTTGAATTCGTGACGAAGAGAACGCTCTCGGACCAATG |
| Up_del_glsB_R | CTGGTCGCCATCAGCGCCCCCAATCGGGAACCGTCTACTGTAGCCAG |
| Down_del_glsB_F | GATTGGGGGCGCTGATGGCGACCAGTGGTATGTAC |
| Down_del_glsB_R | GCTTCTGCAGGTCGACCAAGATAGCATGTTCGCTGACAGCGGC |
| N20_del_glnB_F | ACCATATACTCCGCGCCGGGTTTTAGAGCTAGAAATAGCAAGTTAAAATAAGGC |
| N20_del_glnB_R | GCGCGGAGTATATGGTGGGCTAGCATTATACCTAGGACTGAGCTAGCTGTCAAGG |
| Up_del_glnB_F | tgctttttttgaattcCGCGAAGATCTCTATTACCGCCTCAACGTTG |
| Up_del_glnB_R | CGTCCTCTTCACACTAGTCGCCATGGCAGTGCTTCGCGGACATCGTCCAG |
| Down_del_glnB_F | gcactgccatggcgactagtgtgaagaggacgacgcggcaatttaaacc |
| Down_del_glnB_R | gcttctgcaggtcgacGGTGGCGCAGTGGCAGAATAC |
| N20_del_glnE_F | TGCTCAGTGAATTCCCCGGGTTTTAGAGCTAGAAATAGCAAGTTAAAATAAGGC |
| N20_del_glnE_R | GGGAATTCACTGAGCAATGCTAGCATTATACCTAGGACTGAGCTAGCTGTCAAGG |
| Up_del_glnE_F | tgctttttttgaattcGTGGTTTGACCGACAATATCAAACTCGGC |
| Up_del_glnE_R | GGTAGCGACTAGTCGCCATGGCGACAGCGTAACGGCAGCGTCTTCAC |
| Down_del_glnE_F | cgctgtcgccatggcgactagtCGCTACCGGTGATGAAAGTGAGCGATCACTTAAC |
| Down_del_glnE_R | gcttctgcaggtcgacGTTGTCTGACCAGCGCGTTAACTTCGG |
| N20_del_carAB_F | AGTGGTCGAACAACGGCGGGTTTTAGAGCTAGAAATAGCAAGTTAAAATAAGGC |
| N20_del_carAB_R | CCGTTGTTCGACCACTTTGCTAGCATTATACCTAGGACTGAGCTAGCTGTCAAGG |
| Up_del_carAB_F | TGCTTTTTTTGAATTCCAAGGTCTGGTGATTCGCGACCTGC |
| Up_del_carAB_R | CCCAGTGAATCGGCTCGCGTCCACCGCAAAACCGTGGTTCTG |
| Down_del_carAB_F | GCGGTGGACGCGAGCCGATTCACTGGGAAGTTGTACGC |
| Down_del_carAB_R | GCTTCTGCAGGTCGACCGGGTTCACCGCAAACTGAACGTTGG |
| N20_del_xylAB_F | TCAGGAACATCATTTGTCTCGTTTTAGAGCTAGAAATAGC |
| N20_del_xylAB_R | AAATGATGTTCCTGATAGTATTATACCTAGGACTG |
| Up_del_xylAB_F | GAGTCGACCTGCAGAATGCAAGCCTATTTTGACCA |
| Up_del_xylAB_R | CACAGACATTAAATGGGTATTTAACAGCGTTTCGT |
| Down_del_xylAB_F | CATTTAATGTCTGTGATGCT |
| Down_del_xylAB_R | AGGGTAATAGATCTATTACGCCATTAATGGCAGAA |
| N20_del_nhoA_F | GGAAACTAAACGAATCGGCGGTTTTAGAGCTAGAAATAGCAAGTTAAAATAAGGCTAGTCCG |
| N20_del_nhoA_R | CGCCGATTCGTTTAGTTTCCACTCGTATTATACCTAGGACTGAGCTAGCTGTCAAGGATCCAG |
| Up_del_nhoA_F | TGCTTTTTTTGAATTCTTTCAGGCACAGCGGTGATTTT |
| Up_del_nhoA_R | GGTGATAGCCCGTCGACTAGTTTTCGCCATCATTTGCTGATGTG |
| Down_del_nhoA_F | CAAATGATGGCGAAAACTAGTCGACGGGCTATCACCAGTTTCTC |
| Down_del_nhoA_R | TAATAGATCTAAGCTTCATGGTGTAATTCTATGGGGATGAATCG |

Table S3. pABA production in previous reports

| Compound | Titer (g/L) | Yield(g/g-carbon source) | Host strain | Reference |
| --- | --- | --- | --- | --- |
| pABA | 4.8 | 0.21 (mol/mol) | *E. coli* | [9] |
| pABA | 2.88 | - | *E.coli* | [23] |
| pABA | 0.836 | - | *E. coli* | [30] |
| pABA | 2.46 | 0.16 (mol/mol) | *E. coli* | [11] |
| pABA | 43.1±1.2 | 0.197 (mol/mol) | *C. glutamicum* | [10] |
| pABA | 0.215 | 0.0264 (g/g) | *S. cerevisiae* | [3] |
| pABA | 8.22 | 0.23 (g/g) | *E. coli* | This study |
